# Possible Clues for Brain Energy Translation via Endolysosomal Trafficking of APP-CTFs in Alzheimer's Disease

**DOI:** 10.1155/2018/2764831

**Published:** 2018-10-21

**Authors:** Senthilkumar Sivanesan, Ravi Mundugaru, Jayakumar Rajadas

**Affiliations:** ^1^Department of Research and Development, Saveetha Institute of Medical and Technical Sciences, Chennai 602 105, India; ^2^Biomaterials and Advanced Drug Delivery Laboratory, Stanford University School of Medicine, Stanford, CA 94305, USA; ^3^Department of Bioengineering and Therapeutic Sciences, Schools of Pharmacy and Medicine, University of California San Francisco, San Francisco, CA 94158, USA

## Abstract

Vascular dysfunctions, hypometabolism, and insulin resistance are high and early risk factors for Alzheimer's disease (AD), a leading neurological disease associated with memory decline and cognitive dysfunctions. Early defects in glucose transporters and glycolysis occur during the course of AD progression. Hypometabolism begins well before the onset of early AD symptoms; this timing implicates the vulnerability of hypometabolic brain regions to beta-secretase 1 (BACE-1) upregulation, oxidative stress, inflammation, synaptic failure, and cell death. Despite the fact that ketone bodies, astrocyte-neuron lactate shuttle, pentose phosphate pathway (PPP), and glycogenolysis compensate to provide energy to the starving AD brain, a considerable energy crisis still persists and increases during disease progression. Studies that track brain energy metabolism in humans, animal models of AD, and *in vitro* studies reveal striking upregulation of beta-amyloid precursor protein (*β*-APP) and carboxy-terminal fragments (CTFs). Currently, the precise role of CTFs is unclear, but evidence supports increased endosomal-lysosomal trafficking of *β*-APP and CTFs through autophagy through a vague mechanism. While intracellular accumulation of A*β* is attributed as both the cause and consequence of a defective endolysosomal-autophagic system, much remains to be explored about the other *β*-APP cleavage products. Many recent works report altered amino acid catabolism and expression of several urea cycle enzymes in AD brains, but the precise cause for this dysregulation is not fully explained. In this paper, we try to connect the role of CTFs in the energy translation process in AD brain based on recent findings.

## 1. Introduction

Alzheimer's disease (AD), a progressive neurodegenerative disorder, evolves over many years and is characterized by episodes of memory impairments, loss of cognitive skills [1], and personality changes [2]. Although both tau and amyloid beta (A*β*) reportedly play normal functions at the synapse, the transsynaptic spread of pathogenic tau aggregates and accumulation of toxic A*β* oligomers are together believed to be crucial for synapse loss and neurodegeneration in AD [3]. Despite marked neuronal death during late AD, it is worthwhile to examine the survival of a limited number of A*β*-resistant neurons via increased glucose uptake [4]. Unfortunately, it is difficult to understand how and why only certain nerve cells become resistant to A*β* toxicity, a fact that emphasizes the need to identify precise therapeutic targets for AD. Notably, the toxicity of A*β* oligomer species occurs within seconds to minutes, although it takes several years to attain disease severity. This inequality, along with disparate results of antiamyloid clinical trials, questions the centrality of A*β* as the chief mediator of neuronal cell death [5]. Moreover, whether A*β* oligomeric species directly cause AD is still arguable [6].

Early stages of AD include white matter changes that involve pericyte degeneration and vascular defects with loss of myelinated axons and oligodendrocytes [7]. Hypometabolism occurs early in AD progression, with oxidative stress and impaired mitochondrial bioenergetics [8]. Disruption of normal A*β* synaptic signaling and nonfulfillment of synaptic energy demands presumably cause amyloid toxicity and metabolic stress [5, 9]. Currently, the amyloid cascade hypothesis accentuates the role of soluble A*β* oligomers [10–12] and tau aggregates [13, 14] in AD pathogenesis. However, notable emerging views on the role of insulin resistance and hypometabolism connected to AD suggest that the damage induced by pathogenic A*β* entities could be a secondary effect rather than the early and precise cause of AD.

As part of normal aging, oxygen and glucose metabolic rates are consistently altered in brain cells [15] and are drastically changed in many neurodegenerative diseases [16]. Although numerous alternative pathways could fulfill brain energy needs, there will still be an unmet energy demand [17]. Adenosine triphosphate (ATP) production could be age dependently decreased owing to poor nutrient and oxygen supply as well as reduced rates of glycolysis and oxidative phosphorylation [18]. Brain hypoperfusion and loss of blood-brain barrier (BBB) integrity can diminish nutrient import and/or toxin removal [19]. Recent research focuses on AD hypometabolism-associated cognitive impairments. Reduced glucose transporter 1 (GLUT1) levels cause an age-dependent decrease in cerebral capillary density, reduced cerebral blood flow and glucose uptake, and increased BBB leakage [20]. Indeed, these metabolic and vascular alterations precede dendritic spine loss in cornu ammonis 1 (CA1) hippocampal neurons, and associated behavioral impairments implicate energy dysfunctions during the course of AD. Several seminal works also implicate alterations in spine shape, density, and size of aged neurons, indicative of gross changes in dendritic structures [18].

**Figure 1:**
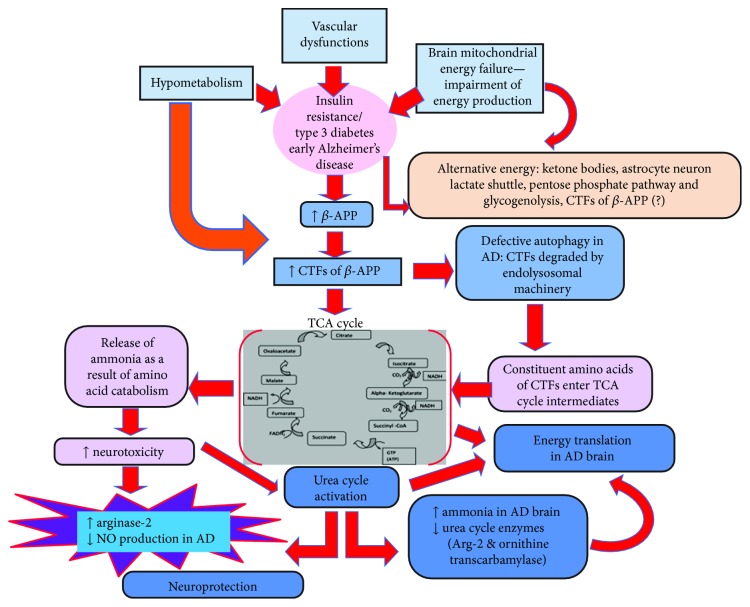
Hypothetical scheme that shows the possible energy compensatory mechanism in hypometabolic AD brain through endolysosomal trafficking of CTFs and urea cycle activation.

The precise cellular events of hypometabolism associated with AD progression are poorly understood. Unfortunately, apart from ketone bodies, astrocyte-neuron lactate shuttle, the pentose phosphate pathway (PPP), and glycogenolysis, there are no clear-cut data that support other energy compensatory mechanisms to balance the bioenergetic deficits in AD brain. Several findings substantiate that plasma levels of certain amino acids are significantly altered in mild cognitive impairment (MCI) and AD patients compared to control individuals [21–23]. The BBB restricts the entry of glutamate and other anionic excitatory amino acids from the circulation [24], although it allows selective transport of certain amino acids to support neuronal functions through transporters [25, 26]. However, whether transported amino acids could support brain energy functions during starvation/hypometabolism is not clear. A growing body of evidence suggests that starvation/hypometabolism in AD brain increases endosomal-lysosomal trafficking of *β*-amyloid precursor protein (*β*-APP) meant for clearance during autophagy. The intricate role of such events and further functions need to be further explored. Moreover, the apparent cause for abnormal *β*-APP processing in AD brains is relatively unclear. This review envisages the importance of vascular abnormalities, hypometabolism, insulin resistance, and altered *β*-APP processing in AD along with our hypothetical views on possible energy compensatory mechanisms in AD brain (Figure 1).

### 1.1. Hypoxia and Hypometabolism Are Early Events in AD

Cerebral amyloid angiopathy- (CAA-) associated microbleeds are one of several causative factors attributed to brain hypometabolism and atrophy in AD [27]. Vascular risk factors, such as adversely affected hippocampus microvasculature length [28], hypoperfusion [29], hypoxia, and hypometabolism, contribute greatly to early AD progression [30–34]. A stronger correlation seems to exist between brain energy inhibition and AD severity [35, 36]. Consistently, agents used to alleviate brain energy dysfunctions are promising for the treatment of cognitive and neurological diseases including AD [37–40].

While fludeoxyglucose positron emission tomography (^18^F-FDG-PET) helps to detect the cerebral metabolic rate of glucose metabolism (MRglc) in AD [41], voxel-based morphometry (VBM) of T1-weighted magnetic resonance imaging (MRI) could reveal brain atrophy [42]. Recent ^18^F-FDG-PET studies confirm hypometabolism in early sporadic AD [43, 44]. Moreover, region-specific severe hypometabolism in AD brain regions shows age invariance with greatly reduced frontal cortex glucose metabolism [45, 46]. There is a strong correlation between glucose hypometabolism and atrophy in the precuneus in early AD subjects based on ^18^F-FDG-PET studies [47]. In mild AD subjects, there is significantly lower MRglc in the parietal cortex, posterior cingulate, and thalamus [48]. Different research groups report hypometabolism in AD/probable AD subjects in the temporal cortex, bilateral middorsolateral frontal region, frontal brain, and parieto-mesial cortex regions based on ^18^F-FDG-PET [42, 49, 50]. Although Stein et al. [51] identified energy fluctuations manifested as poor glucose metabolic rate in the limbic areas of the temporal lobe, a voxel-based study that analyzed AD brain regions revealed significant atrophy of the hippocampus and amygdala [52]. Metabolic fluctuations of key glycolytic enzymes [53] and oxygen delivery in the circulating erythrocytes could also be risk factors for brain hypometabolism in AD [54].

The “Warburg effect,” well described for cancer cells, appears to apply to neuronal cells [4], notably during the mild phase of AD where neurons with defective glucose uptake show resistance to A*β* toxicity. Thus, the predementia phase of AD provides evidence of gray matter loss and brain glucose deficits [55]. Reduced expression of energy metabolism genes that encode subunits of the mitochondrial electron transport chain is also apparent in the posterior cingulate neurons of AD brains [56]. Mitoenergetic failure develops in A*β* overexpressing *Caenorhabditis elegans*, a finding that demonstrates this phenomenon is not unique to human AD [57]. Using Trem2^−/−^ 5xFAD transgenic mice, mutations in triggering receptor expressed on myeloid cells 2 (TREM2) can lead to energy dysfunctions in the immune cell microglia and thereby cause impaired clearance of amyloid plaques; energy supplementation with cyclocreatine to immune cells reduces the plaque load to protect the neurons [58]. Altogether, vascular and metabolic fluctuations are well indicated during the early course of AD.

### 1.2. Defective Glycolytic Enzymes, Glucose Transporters, and Impaired Insulin Signaling in AD

It is difficult to determine whether brain hypometabolic status is a consequence or cause of AD pathology, but many recent works support the latter. Elevated plasma glucose concentrations in fasting conditions are attributed to increased brain glucose levels in AD, a finding that implicates insulin resistance [59]. Human and animal studies clearly show reduced expression of glucose transporters in aged [60] and AD [59] brains, including changes in the expression of key enzymes involved in glycolysis and oxidative phosphorylation [61–63]. Evidence supports considerable neuronal loss in AD brain that involves oxidative stress-mediated inhibition of glyceraldehyde-3-phosphate dehydrogenase (GAPDH) activity [64]. The reduced flux of pyruvate carboxylase (PC) in gluconeogenesis and pentose phosphate from the hexose monophosphate shunt reported in the brains of A*β*PP-PS1 mice (an AD model) further indicates poor glucose metabolism [65]. A study with Thy-1 mito-CFP mice showed reduced ATP levels in white matter during aging, in correlation with ultrastructural alterations in mitochondria, as well as reduced association of mitochondria with the endoplasmic reticulum [66]. The van Gijsel-Bonnello et al. group [67] recently performed metabolomic analysis using 5xFAD transgenic mouse astrocytes and showed marked changes in the glycolytic pathway and tricarboxylic acid cycle (TCA).

A considerable number of research substantiates glucose transporter defects [20, 68–70], neurovascular dysfunctions associated with glucose transporter defects [20], poor glucose utilization [71, 72], and cognitive dysfunctions [73] in AD. The defective insulin signaling mechanisms reported in AD [70, 74–77] further strengthen the vascular and metabolic anomalies in AD, and the disease could be described as type 3 diabetes [78, 79]. Therefore, therapeutics aimed towards curing AD would need to significantly rely on regulating insulin levels and brain cellular energy levels [80–84]. Insulin improves cognition and may be neuroprotective, yet different intranasal insulin concentrations exert varying responses in subsets of AD patients [39, 40, 84–86].

### 1.3. Hypoxia and Energy Stress/Starvation Influences *β*-APP Processing and Beta-Secretase 1 (BACE1) Levels

Defective glycolysis [59] and oxidative phosphorylation tend to be part of the metabolic adaptation process implicated as early signs of sporadic AD [15]. Substantial evidence pinpoints that hypoxia significantly increases BACE1 gene expression through hypoxia-inducible factor 1-alpha (Hif1*α*) upregulation [32, 87–89]. Although increased BACE1 activity reduces mitochondrial glucose uptake [90], a majority of work supports the view that BACE1 upregulation accounts for energy inhibition [36, 91–93]. Gabuzda et al. [94] explored energy-related metabolic stress on APP processing using sodium azide and agents that inhibit protein transport in the secretory pathway (monensin and brefeldin A). These agents could ultimately drive oxidative energy impairment in mitochondria to alter several-fold APP proteolytic processing into an 11.5 kDa carboxy terminal fragment. In another work, Velliquette et al. [91] revealed that energy inhibition by agents such as insulin, 2-deoxyglucose, 3-nitropropionic acid, and kainic acid in wild-type and Tg2576 transgenic mice strikingly elevates cerebral BACE1 levels concomitant with progressive AD pathology. Xiong et al. [93] inhibited mitochondrial complex I, II, and IV with rotenone, nitropropionic acid, and sodium azide, respectively, to prove that mitochondrial respiratory inhibition and oxidative stress trigger BACE1 expression as well as the presence of *β*-APP carboxy terminal fragments. Gatta et al. [92] inhibited heme synthesis and mitochondrial energy production using small interfering RNA and N-methylprotoporphyrin IX; these actions alter APP processing and amyloid aggregation. In another work [95], fasting differentially activates macroautophagy in 5xFAD mouse neurons compared to control mice. Fasting alters the numbers and pattern of autophagosomes in neurons, although the study suggests that activated macroautophagy after fasting does not degrade intracellular A*β* that is increased due to enhanced uptake from the extracellular space [95]. Inhibition of the key glycolytic enzyme 6-phosphofructo-2-kinase in astrocyte cultures increases amyloidogenic processing of APP [96]. Unfortunately, the cause for increased BACE1 and *β*-APP processing in conditions such as energy inhibition, starvation, and hypometabolism in AD brain is still unclear. However, there is a plausible relationship between hypometabolism and autophagy. Specifically, defects in mammalian target of rapamycin (mTOR) signaling in AD brain correlate with impaired mitochondrial functions and energy metabolism [97]. Whether hypometabolism precedes defective autophagy or the vice versa requires further exploration.

### 1.4. Connection between Early Autophagy/Macrophagy and *β*-APP Processing

Macroautophagy activation occurs during conditions such as cellular stress due to nutritional deficit or cell injury [98]. This process represents a lysosomal pathway for the turnover of organelles and long-lived proteins and is a key determinant of cell survival and longevity [99]. While chaperone-mediated autophagy (CMA) involves chaperone-mediated degradation of proteins near the lysosomal lumen that have specific sequence signals [100], macroautophagy degrades misfolded and aggregated proteins by lysosomal machinery [101]. Using a fusion protein called tandem-fluorescent-APP, investigators showed that starvation triggers the trafficking of APP and APP-carboxy-terminal fragments (APP-CTFs) to the degradative endolysosomal network, a finding that provides a new hint for identifying key therapeutics for AD [102]. A more recent work indicates increased BACE1 turnover in the vicinity of suppressed autophagy after lysosomal inhibition [103]. Since BACE1 recruitment to the autophagy pathway and its comigration with autophagic vacuoles seems to occur similarly along the entire axon, autophagic induction with concomitant BACE1 retention could promote increased *β*-APP cleavage processing. Taken together, the protective role of autophagy elicited under metabolic stress conditions reveals bioenergetic adaptations in the cellular environment [104]. Thus, autophagy could provide compensatory regulation of brain energy through the APP trafficking pathway.

Culminating evidence pinpoints early induction of neuronal macroautophagy in sporadic AD brains and transgenic mouse models of AD pathology even before the visualization of extracellular A*β* deposits [99, 105]. While dystrophic dendrites are the sites where autophagosomes and late autophagic vacuoles (AVs) accumulate, it is possible that purified AVs are the source of APP, beta-cleaved APP, presenilin 1, nicastrin, and *γ*-secretase activity. Nonetheless, Boland et al. [106] reveal that although neuronal macroautophagy does not directly regulate APP metabolism, there is plausible proof for an antiamyloidogenic role of lysosomal proteolysis in postsecretase APP-CTF catabolism. Indeed, agents/drugs that can reduce A*β* levels in the brain probably account for the increased degradation of APP-CTFs as well as A*β* clearance [107].

### 1.5. Amino Acid Sensing in Lysosomes Provides Hints about the Brain Energy Translation Process

The role of lysosomal machinery in the degradation and recycling of cellular waste is not novel. Moreover, the localization of lysosomal hydrolases within neurons is a feature of AD [108]. However, compelling evidence supports additional lysosomal machinery functions involved in secretion, plasma membrane repair, and energy metabolism [109]. While several studies provide mechanistic insights about the lysosome and cellular energy metabolism [109–112], much remains to be explored in brain regions and particularly in AD. Other studies show a favoring of amino acid sensing and mTOR signaling promoted by vacuolar H^+^-ATPase within the lysosomal compartment [113–115]. In a recent review, Carroll and Dunlop [116] detail the role of lysosomes in autophagy, in amino acid sensing by mTOR complex 1 (mTORC1), and the key signaling events associated among lysosomes, adenosine monophosphate-activated protein kinase (AMPK), and mTORC1. Considering this point of view, the events associated with endosomal-lysosomal trafficking of *β*-APP [102, 117] need much more exploration to support energy-related functions of cleaved APP fragments.

### 1.6. Culminating Work Shows Amino Acid Catabolism and Urea Cycle Activation in AD Brain

While ketone bodies [118, 119], PPP [67], and astrocyte-neuron lactate shuttle [120] try to compensate for AD brain energy deficits, there is still (to some extent) an increasing demand for brain energy. Urea cycle activation in AD brain [121–123] to remove ammonia generated from increased amino acid catabolism [121, 123, 124] provides evident and plausible insights into amino acid catabolism for energy conversion. AD brains exhibit increased expression of carbamoyl phosphate synthetase 1 (CPS-1) and peptidylarginine deiminase (PAD), which catalyzes the conversion of arginine into citrulline during ammonia formation [124]. The normal brain lacks urea-cycle-related ornithine transcarbamylase (OTC) and CPS-1 enzymes, a finding that strengthens the fact that AD brains are vulnerable to ammonia toxicity. Favorably, defects in ammonia detoxification owing to the adversely affected energy-producing pathway of glucose metabolism (involving PC and PPP) were reported in 20-month-old A*β*PP-PS1 mice [65].

According to Seiler [125], hyperammonemia in the blood and brain of AD subjects can provoke toxicity, contribute to AD pathogenesis, and alter *β*-APP processing in the lysosome. Ammonia is the major end product of cellular amino acid metabolism [126], and the brain can derive ammonia from both endogenous and exogenous pathways [125, 127]. Endogenous sources include degradation of neurotransmitters (glutamate, aspartate, and other monoamines), amino acids (e.g., glutamine, asparagine, and glycine), and hexamines. Deamination of amino-purines, amino-pyrimidines, and oxidative deamination of primary amines overall contribute to the endogenous sources of brain ammonia. Given the fact that the urea cycle in AD brains generates copious amounts of ammonia, a considerable amount of protein degradation must occur during AD pathogenesis. Increasing evidence pinpoints amino acid catabolism [128, 129] and ammonia accumulation in AD brain [128, 130, 131], although the exact processes remain unclear. However, such notable findings could indicate activation of energy compensation regulatory mechanisms in hypometabolic AD brain. Findings from Hansmannel et al. [121] strikingly reveal that all urea cycle enzymes are expressed in AD brain, with notably increased arginase-2 (Arg2) gene expression compared to control. Moreover, vulnerable AD brain regions exhibit region-specific alterations in arginase and nitric oxide synthase (NOS) [132]. OTC induction and Arg2 upregulation in AD would attempt to reduce brain ammonia, including reduction of NO and inducible NOS levels, actions that may emphasize the intricate neuroprotective role of the urea cycle pathway [121]. In Huntington's disease (HD), another neurodegenerative disease, markedly elevated toxic urea levels in the brain before dementia onset precede brain damage in a transgenic sheep HD model and human subjects [133]. Since urea and ammonia are the key metabolic units of protein catabolism, there is a clear-cut indication for major protein catabolism in neurodegenerative HD and AD. Interestingly, a recent ongoing phase 2 clinical trials for AD is testing AMX0035 which is an oral formulation of two drugs, sodium phenylbutyrate (PB) and tauroursodeoxycholic-acid (TUDCA). While PB is an FDA-approved drug prescribed for urea-cycle disorders to reduce toxic unfolded proteins, TUDCA helps in reducing cellular energy loss.

### 1.7. Evidence Spotlights the Importance of APP-CTFs-Derived Amino Acids in Brain Energy Functions in AD

**Figure 2:**
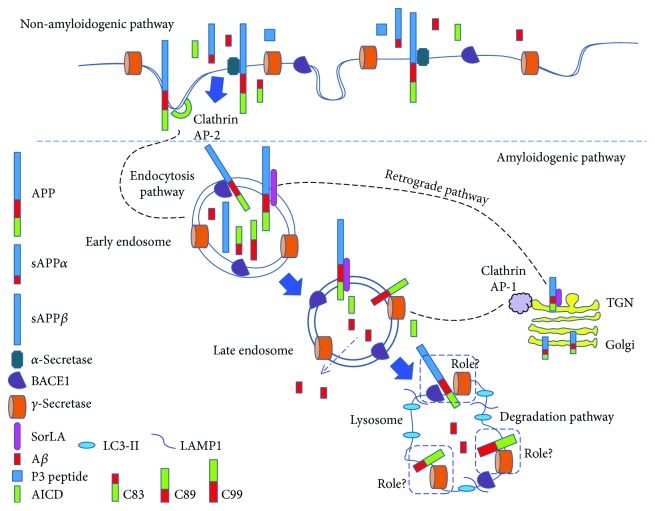
Pathways involved in the proteolytic processing of *β*-APP in AD.

Serum and cerebrospinal fluid metabolomics studies reveal major alterations in canonical energy metabolism pathways, Krebs cycle, mitochondrial function and amino acid metabolism in MCI and AD patients [22]. Since starvation promotes trafficking of both APP and APP-CTFs to the degradative endolysosome [102], it provides clues for compensatory mechanisms initiated to counterbalance the energy deficits. Proteolytic processing of APP mainly involves amyloidogenic and nonamyloidogenic pathways [134, 135] as detailed below (Figure 2). BACE1 cleavage of APP within its extracellular/luminal domain represents the amyloidogenic pathway and promotes shedding of soluble *β*-secreted APP (sAPP*β*) and membrane-associated C-terminal fragment of 99 amino acids (*β*CTF/C99) or 89 amino acids (*β*CTF/C89). After ectodomain shedding, *γ*-secretase (protein complex comprised of presenilin 1 or 2, Aph1 homolog A or B, nicastrin, and presenilin enhancer protein 2) cleavage occurs in the membrane-tethered C89 and C99 and thereby releasing p3 from C89 or A*β* peptides from C99. The nonamyloidogenic pathway at the plasma membrane involves cleavage by *α*-secretases (ADAM10/ADAM17) that generates soluble *α*-secreted APP (sAPP*α*) and a C-terminal membrane-bound fragment of 83 amino acids (*α*CTF/C83). Further cleavage of this fragment by *γ*-secretase releases the p3 peptide. The retrograde trafficking mechanisms that follow either *α*- or *β*-secretase cleavage allow delivery of APP-CTFs to the trans-Golgi network (TGN). Based on recent findings, the majority of *γ*-secretase cleavage of APP occurs in the TGN [136]. Notably, *γ*-secretase cleavage of both *β*CTF/C99 and *α*CTF/C83 generates the APP intracellular domain (AICD), but it is rapidly degraded similar to p3. Growing evidence depicts novel/alternative metabolic processing pathways (*η*-secretase, *δ*-secretase, and meprin pathways) during physiological processing of APP [137, 138]. Usually, carboxy-terminal fragments, such as *β*CTF/C89, *β*CTF/C99, and *α*CTF/C83, are targeted to the endosomal-lysosomal compartment [102, 139]. The biological importance of *β*CTF/C99, *β*CTF/C89, *α*CTF/C83, and p3 remains unclear [135], although many studies substantiate the neuroprotective functions of sAPP*α*, including in brain development, growth factor, neural cell proliferation [140, 141], and synaptic plasticity [142]. sAPP*β* is involved in pruning of synapses during the development of both central and peripheral neurons [143] but reported to cause suppression of neuronal stem cell differentiation [144]. Although controversies exist about the biological functions of the AICD fragment, it is reported to be involved in gene transcription, cytoskeletal dynamics, and apoptosis [145]. However, during lysosomal degradation that recruits various CTFs (*β*CTF/C89, *β*CTF/C99, and *α*CTF/C83), it is likely that constitutive amino acids formed after proteolytic cleavage could serve as TCA cycle intermediates to fulfill brain energy deficits [146]. It remains elusive whether cleaved amino acids from APP after degradation in endolysosomes [102] also provide energy via the TCA cycle. This could be due to the fact that during APP sorting, beclin1 promotes targeting of a smaller fraction of surface-internalized APP to microtubule-associated protein 1 light chain 3- (LC3-) positive phagophores for degradation [147]. According to Hoyer et al. [148], hypoxia/sodium azide insults in HEK293 cells stably transfected with bAPP695 show energy failure with strikingly elevated ATP turnover and adenosine levels that parallel intracellular APP increases. In another work, upon hypoglycemic induction, rat neuronal cultures increase utilization of amino acids as evidenced by ammonia formation due to amino acid catabolism [149]. Overall, there is a considerable body of evidence that supports *β*-APP energy-related functions during AD hypometabolism. Further detailed investigations are warranted on APP-related energy functions in order to accurately track the pathological cascade to develop precise therapeutic targets. Recently, a study revealed that a greater number of dendritic spines in healthy brains (controls) compared to age-matched AD brains poorly correlate with symptoms such as dementia in controls, despite marked amyloid plaques and tangles observed in both types of brains [150]. This intriguing result demarcates the disease and healthy brains from the amyloid cascade hypothesis and creates avenues for further exploration in AD research.

## 2. Summary

AD brain is vulnerable to increased *β*-APP proteolytic cleavage and accumulation of various CTFs as well as APP in the endolysosomal compartment. While these intricate events are complicated, the relative cause and functions of cleavage products are poorly understood. In this paper, we hypothesize that in hypometabolic AD brains, the constitutive amino acids of CTFs and APP formed during endolysosomal degradation could compensate for metabolic demands through the TCA cycle. Our hypothetical views on APP-related brain energy functions can be justified with numerous recent works that implicate amino acid catabolism, urea cycle activation (due to increased amino acid catabolism), and ammonia toxicity propagated in AD brain. We also try to connect possible neuroprotective mechanisms in AD brain against ammonia toxicity through activation of urea cycle enzymes (Arg-2 and OTC) and decreased nitric oxide (NO) levels.
